# Molecular Mechanisms of Resistance to Cyhalofop-Butyl in Barnyard Grass (*Echinochloa crus-galli*)

**DOI:** 10.3390/plants15121908

**Published:** 2026-06-19

**Authors:** Guangyu Li, Yuanju Huang, Yongjie Yang, Suxin Zhang, Chun Wang, Qian Wang, Mingjia Sun

**Affiliations:** 1 Institute of Plant Protection, Heilongjiang Academy of Agricultural Sciences, Harbin 150086, China; guangyl2022@163.com (G.L.); 18846810430@163.com (S.Z.); chunharbin@aliyun.com (C.W.); wangqian999@126.com (Q.W.); 2 State Key Laboratory of Rice Biology and Breeding, China National Rice Research Institute, Hangzhou 311400, China; 3 Beidahuang Kenfeng Seed Industry Co., Ltd., Harbin 150006, China; 17766514757@163.com

**Keywords:** cyhalofop-butyl, *Echinochloa crus-galli*, target-site resistance (TSR), non-target-site resistance (NTSR), transcriptomics

## Abstract

Cyhalofop-butyl, a widely used acetyl-CoA carboxylase (ACCase)-inhibiting herbicide, is increasingly resisted by *Echinochloa crus-galli* in paddy fields. However, the physiological and molecular mechanisms of this resistance have not been fully elucidated. In this study, a highly resistant population (HL-R, resistance index [RI] = 19.05) from Hulin and a susceptible population (HL-S, RI = 1.00) were used. The results showed that HL-R population exhibited broad-spectrum resistance to five herbicides (RI = 1.98–14.53). No target-site mutations were found in the *ACCase* gene; instead, its overexpression contributed to target-site resistance (TSR). Metabolic inhibitor assays confirmed non-target-site metabolic resistance (NTSR). Transcriptome and qRT-PCR analyses identified two detoxification-related differentially expressed genes (DEGs; *CH01.636*, *BH02.4557*) as key regulators. The activity of the antioxidant enzyme peroxidase (POD) was also associated with resistance. Collectively, the high resistance of HL-R to cyhalofop-butyl is synergistically driven by *ACCase* overexpression, POD activity and P450/GST-mediated metabolic detoxification. These findings provide theoretical guidance for the management of resistant barnyard grass.

## 1. Introduction

Barnyard grass (*Echinochloa crus-galli*) is one of the most pernicious weeds in paddy fields in China [1]. As a C4 plant, it has advantages over rice, a C3 plant, in obtaining light and nutrients [2]. Barnyard grass can cause yield reduction even at a low density [3]. In paddy fields where weed control measures fail, rice yields may decline by 50% or more [4]. The interference of barnyard grass on rice is 2.31-fold greater than intraspecific competition among rice plantlets, driven by extensive root system interactions [5]. In addition, barnyard grass serves as an alternative host for a variety of insect pests and pathogens [6]. Therefore, controlling barnyard grass is an essential precondition in rice cultivation.

Cyhalofop-butyl, a selective and translocated ACCase inhibitor, is commonly used to control barnyard grass [7]. It inhibits ACCase enzymes, thereby disrupting physiological processes and consequently leading to barnyard grass death [8]. However, decades of applications of this herbicide have driven the evolution of resistance in barnyard grass populations, rendering this issue increasingly severe and concerning [9,10]. Therefore, elucidating its resistance mechanisms is important for barnyard grass management.

As summarized, cyhalofop-butyl penetrates the plant, is translocated and accumulates at the action site, binds to target proteins, and alters or destroys biological functions. The process that impairs the binding affinity of herbicides to their target proteins is defined as TSR. TSR is mainly caused by genetic modifications, including point mutations in the target enzyme gene, as well as overexpression or amplification of the target gene while all other factors that lead to herbicide resistance are collectively referred to as NTSR [11]. In weeds resistant to ACCase inhibitors, TSR is mainly caused by gene mutations in the target enzyme, with mutation sites occurring in the coding region of *ACCase* CT domain [12]. To date, seven mutation sites with a total of eighteen amino acid substitution variants have been reported (Ile-1781-Val/Ala/Leu/Thr, Trp-1999-Leu/Cys/Ser, Trp-2027-Leu/Ser/Cys, Ile-2041-Val/Thr/Asn, Asp-2078-Glu/Gly, Cys-2088-Arg, Gly-2096-Ser/Ala) [13,14,15,16,17]. Overexpression of the target enzyme is also a component of the TSR mechanism [9]. NTSR is regarded as an important component of plant stress response, and its molecular mechanism is complex, usually involving the synergistic action of multiple genes from large gene families [18], including cytochrome P450 monooxygenases (CYP450s), glutathione S-transferases (GSTs), glycosyltransferases (GTs), and ATP-binding cassette (ABC) transporters [19]. Nevertheless, the specific TSR and NTSR mechanisms conferring resistance to cyhalofop-butyl in barnyard grass vary among different populations, and further research is needed to fully elucidate the molecular diversity.

In weed science, omics approaches have been widely used to interpret resistance mechanisms [20]. Transcriptomics is a systematic approach that can identify and quantify transcriptomes at the tissue, cellular, or subcellular level, promoting the characterization of DEGs [21]. These approaches were increasingly used to clarify mechanisms in *Conyza canadensis* and *Amaranthus tuberculatus*, and a range of weed species [22,23]. Moreover, herbicide stress induces weeds to produce reactive oxygen species (ROS), which trigger the oxidation of intracellular macromolecules, initiate membrane lipid peroxidation, and accelerate plant cell senescence and death [24]. Malondialdehyde (MDA), a major byproduct of ROS-mediated lipid peroxidation, is widely recognized as a reliable biomarker for quantifying the degree of oxidative damage in plants [25]. The antioxidant enzyme system plays a crucial role in eliminating excessive ROS and reducing herbicide damage in weeds, and increased activity of this system is one of the key determinants underlying the evolution of herbicide resistance in weeds [26]. Research has demonstrated that glyphosate-resistant *Amaranthus palmeri* exhibits enhanced activities of antioxidant enzymes (e.g., superoxide dismutase [SOD] and catalase [CAT]), which synergistically promote the expression of metabolic resistance [27]. In *Sagittaria trifolia*, enhanced antioxidant capacity was the primary mechanism mediating resistance to bensulfuron-methyl [28]. Therefore, alterations in MDA content and antioxidant enzyme activity are of great significance for a comprehensive understanding of the resistance to cyhalofop-butyl in barnyard grass.

In this study, PCR was used to determine the presence of mutations in *ACCase* gene locus and qPCR was used to quantify its expression in the resistant population. Transcriptome sequencing technology was adopted to screen key metabolism-related genes. Physiological and biochemical indices were determined to comprehensively clarify the resistance mechanism of barnyard grass to cyhalofop-butyl, providing a theoretical basis for the control of herbicide-resistant barnyard grass.

## 2. Results

### 2.1. Whole-Plant Dose–Response to Cyhalofop-Butyl and Other Herbicides

**Figure 1:**
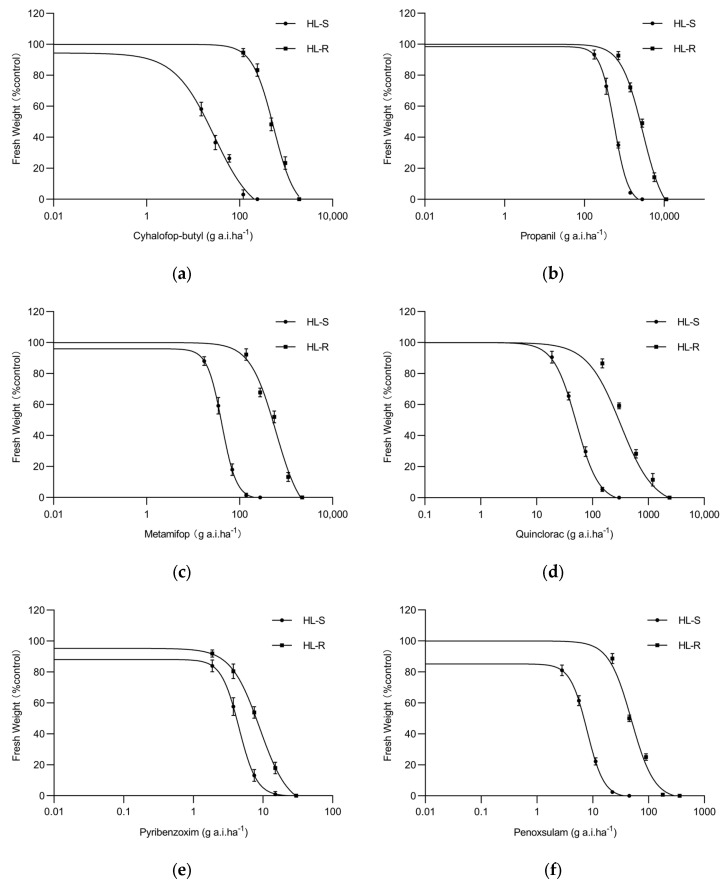
Whole-plant dose–response of HL-S and HL-R populations of *Echinochloa crus-galli* to cyhalofop-butyl (**a**), propanil (**b**), metamifop (**c**), quinclorac (**d**), pyribenzoxim (**e**), and penoxsulam (**f**). Values are mean ± SE of three biological replicates.

The whole-plant bioassay indicated that the *GR_50_* values of HL-S and HL-R against cyhalofop-butyl were 28.33 and 539.8 g a.i.ha^−1^, respectively (Figure 1a). Based on the *GR_50_* values, the RI for HL-R was 19.05 compared to 1.00 for the HL-S population. The *GR_50_* values of HL-S to propanil, metamifop, quinclorac, pyribenzoxim, penoxsulam were 557.0, 41.45, 51.13, 4.525, 7.911 g a.i.ha^−1^, respectively, while those for HL-R were 2864.0, 602.1, 317.5, 8.963, 49.36 g a.i.ha^−1^ (Figure 1b,f), representing 5.14-, 14.53-, 6.21-, 1.98-, 6.24-fold higher resistance compared to HL-S. These findings clarified that HL-R exhibits a high level of resistance to cyhalofop-butyl and shows different levels of resistance to other herbicides, with the lowest resistance level to pyribenzoxim.

### 2.2. ACCase Gene Sequencing and Relative Expression

**Figure 2:**
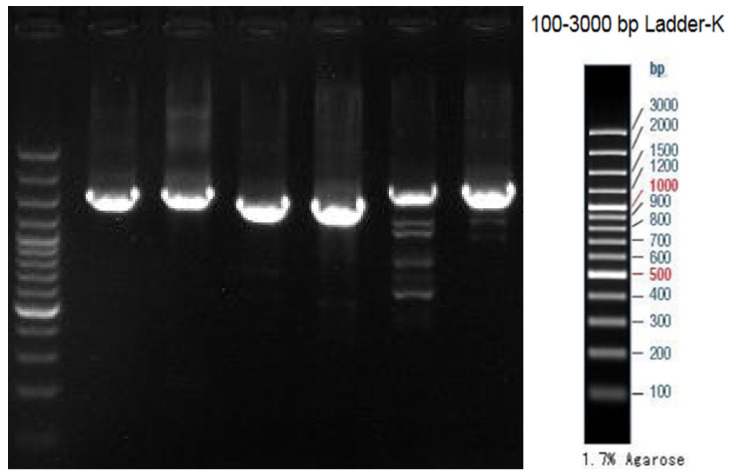
PCR product agarose electrophoresis detection.

**Figure 3:**
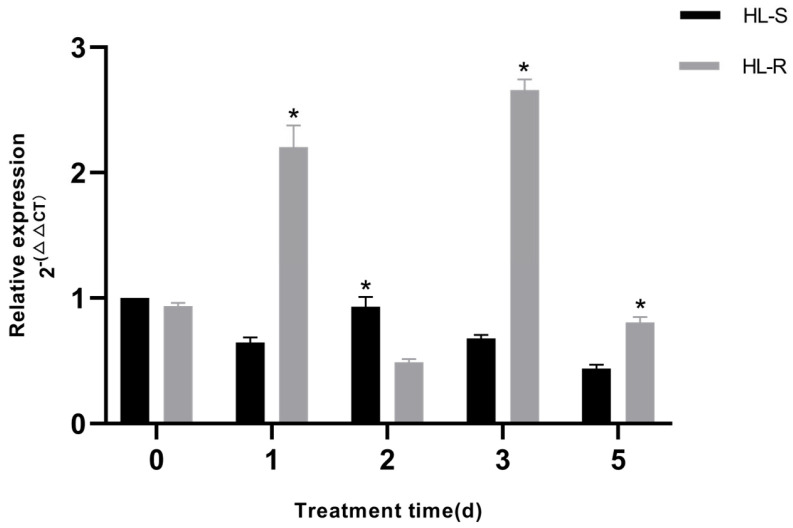
Relative expression of *ACCase* gene treated with cyhalofop-butyl in HL-S and HL-R populations of *Echinochloa crus-galli.* * Significant difference between the two groups (*p < 0.05*).

The target band of approximately 1500 bp was obtained through specific primer amplification (Figure 2). Compared with the seven specific amino acid residue sites of the *ACCase* CT functional region that have been reported, no amino acid changes were found at any of the seven previously reported *ACCase* mutation sites (Ile-1781, Trp-1999, Trp-2027, Ile-2041, Asp-2078, Cys-2088, and Gly-2096) in the HL-S and HL-R populations. Analysis of target gene expression levels revealed highly significant differences between HL-S and HL-R after cyhalofop-butyl treatment (Figure 3). Specifically, the expression level in HL-R was significantly lower than that in HL-S on day 2, but significantly higher on all other days. These findings suggest that the underlying mechanism conferring cyhalofop-butyl resistance in HL-R involves overexpression rather than genetic mutation of the target enzyme.

### 2.3. The Effects of Metabolic Inhibitors on Resistance

The P450 inhibitor malathion and the GST inhibitor NBD-Cl had no significant effect on the *GR_50_* of HL-S. The *GR_50_* of HL-R decreased from 539.8 g a.i.ha^−1^ to 109.2 and 139.0 g a.i.ha^−1^, respectively. Consequently, the RI dropped from 19.05-fold to 3.85-fold and 4.91-fold respectively (Table 1). This indicates that HL-R has metabolic resistance to cyhalofop-butyl mediated by P450 and GST.

### 2.4. Transcriptome Analysis and Identification of DEGs

A total of nine cDNA libraries were constructed in this study. After filtering the original data, there were 42,154,302–66,725,878 valid pieces of data. Taking the *E. crus-galli* [29] genome sequence as the reference genome, sequence alignment was performed. Among the nine clean reads obtained from transcriptome sequencing, the proportion of reads that aligned to a unique position of the reference genome was 88.89–89.86%, and the proportion that aligned to multiple positions of the reference genome was 7.02–7.96%. The Q20 and Q30 for the nine libraries were above 99.25% and 97.31%, respectively, and the GC content was above 55.13% (Table S1). This indicates that the vast majority of reads can be mapped to a unique corresponding sequence in the reference genome, which facilitated the subsequent design of primers and amplification of the screened metabolic difference genes.

**Figure 4:**
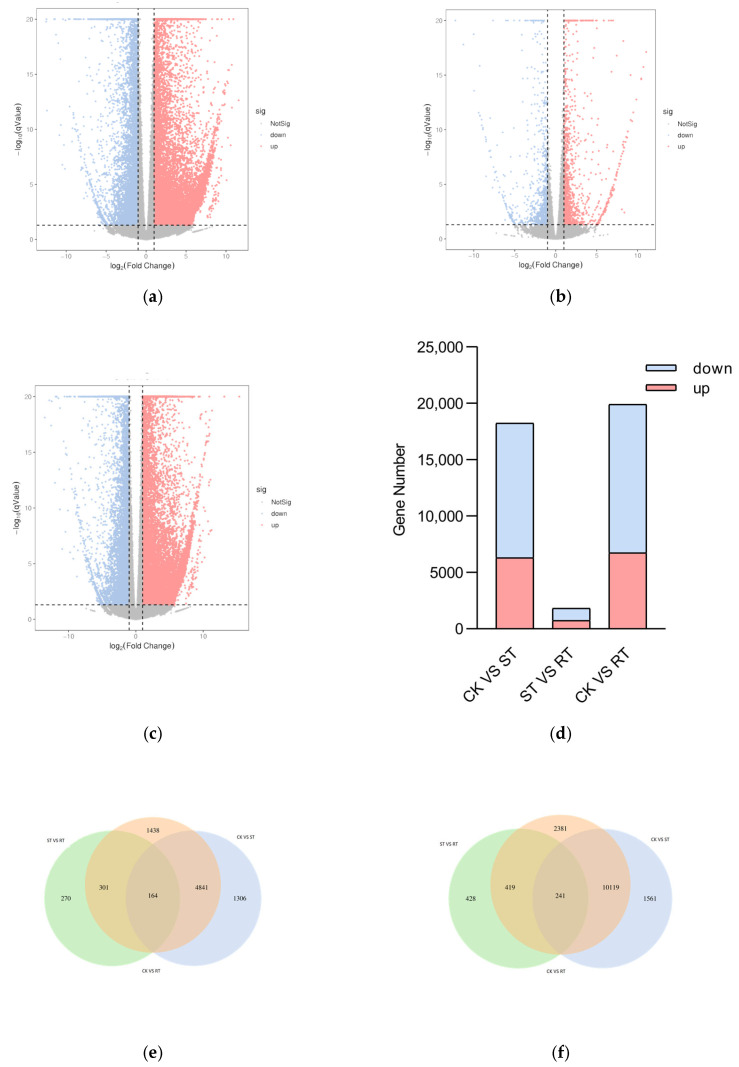
The volcano maps of DEGs. Red represents significantly up-regulated genes, green represents significantly down-regulated genes, and gray represents insignificant genes; (**a**) CK vs. ST, (**b**) ST vs. RT, (**c**) CK vs. RT. (**d**) DEGs in histograms, (**e**) Venn diagram of down-regulated genes. (**f**) Venn diagram of up-regulated genes.

The transcriptome sequencing results indicated that the total number of DEGs in the three comparison groups was 18,232 (CK vs. ST), 1823 (ST vs. RT), and 19,904 (CK vs. RT), respectively (Tables S2–S4). Among these, the CK vs. RT comparison had the most DEGs, while ST vs. RT comparison had the fewest (Figure 4a–c). The numbers of up-regulated genes were 6311, 735 and 6744 respectively, and the numbers of down-regulated genes were 11,921, 1088 and 13,160 respectively (Figure 4d). The Venn diagram showed 241 down-regulated genes and 164 up-regulated genes common to all three comparison groups, while 270 up-regulated genes and 428 down-regulated genes were specific to the ST vs. RT comparison (Figure 4e,f).

### 2.5. GO Enrichment

**Figure 5:**
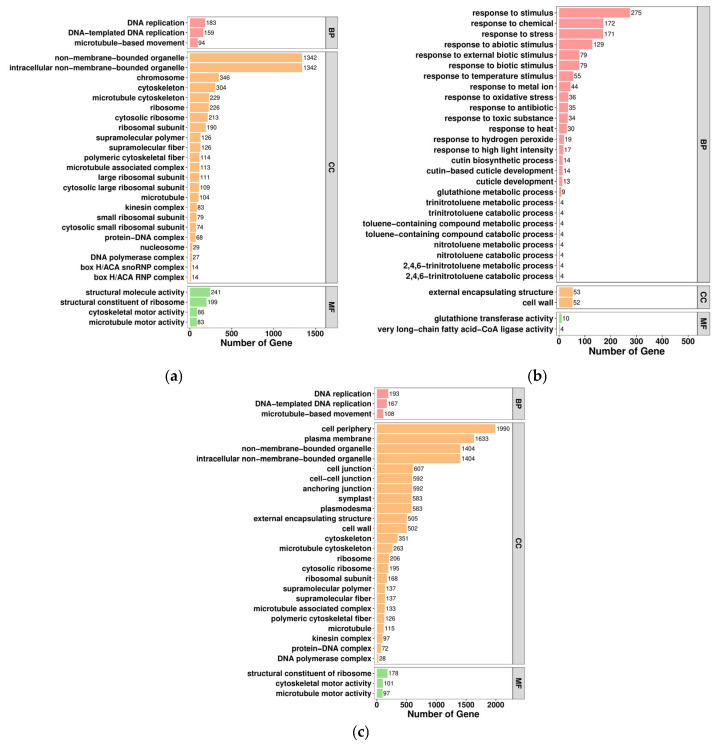
GO annotation classification of DEGs in histograms. The horizontal axis represents the number of genes. The vertical axis represents the functional classification. Red represents biological process, orange represents cellular component, green represents molecular function. (**a**) CK vs. ST, (**b**) ST vs. RT, (**c**) CK vs. RT.

GO enrichment analysis was conducted on the identified DEGs. The GO database includes three ontologies: biological process, cellular component and molecular function. The basic units are terms, each corresponding to a function or attribute. In the CK vs. ST comparison, DEGs were extremely significantly enriched in 512 terms (*p* < 0.01), of which 347 were biological processes, accounting for 67.8% (Table S5). The 30 terms with the most significant enrichment were selected for presentation, including six biological processes, 20 cellular components and four molecular functions (Figure 5a). In the ST vs. RT comparison, DEGs were extremely significantly enriched in 113 terms (*p* < 0.01), of which 58 were biological processes, accounting for 51.3% (Table S6). Among the top 30 terms, there were 26 biological processes, two cellular components and two molecular functions (Figure 5b). In the CK vs. RT comparison, DEGs were extremely significantly enriched in 437 terms (*p* < 0.01), of which 294 were biological processes, accounting for 67.3% (Table S7). Among the top 30 terms, there were five biological processes, 22 cellular components and three molecular functions (Figure 5c).

### 2.6. KEGG Enrichment

**Figure 6:**
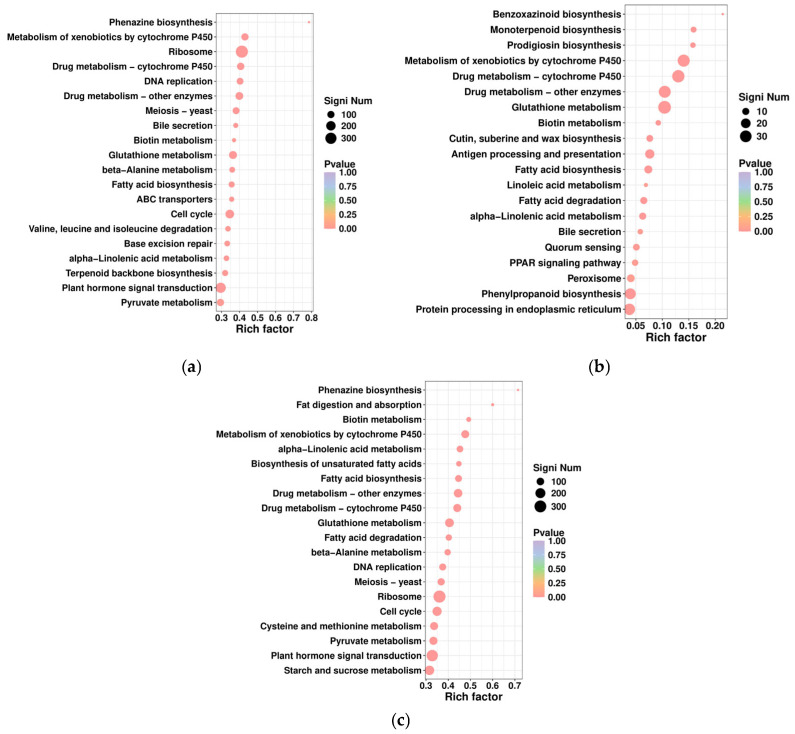
KEGG annotation classification of DEGs in histograms. The horizontal axis represents the number of genes. The vertical axis represents the functional classification. Red represents cellular processes, orange represents environmental information processing, green represents genetic information processing, blue represents metabolism, purple represents organismal systems. (**a**) CK vs. ST, (**b**) ST vs. RT, (**c**) CK vs. RT.

Scatter plots were used to present the KEGG enrichment analysis of DEGs. The degree of enrichment was measured by rich factor, q-value and the number of genes enriched in each pathway. The rich factor refers to the ratio of the number of DEGs enriched in a pathway to the number of annotated genes; a larger value indicates a greater degree of enrichment. The q-value ranges from 0 and 1, with values closer to 0, indicating more significant enrichment. The figures show the 20 most significantly enriched pathways. The most significantly enriched pathways in the CK vs. ST, ST vs. RT, and CK vs. RT comparisons were Ribosome, metabolism of xenobiotics by cytochrome P450, and metabolism of xenobiotics by cytochrome P450, with 364, 33, and 112 genes enriched, respectively (Figure 6). The complete data are presented in (Tables S8–S10).

### 2.7. Quantitative Real-Time PCR Analysis of Genes

**Figure 7:**
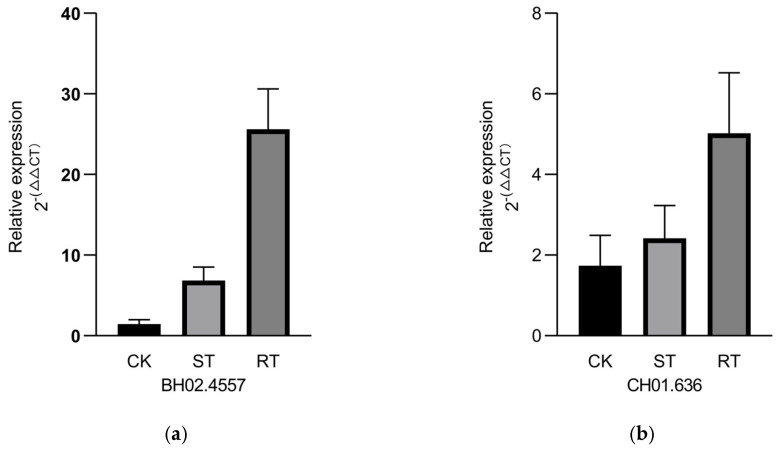
Quantitative real-time PCR analysis of genes. (**a**) The expression levels of *BH02.4557* in different treatments; (**b**) the expression levels of *CH01.636* in different treatments. Values are the mean ± SE of three biological replicates.

Based on the results of the metabolic enzyme inhibitor assays and differences in gene expression levels, this study focused on genes from the P450 and GST enzyme families related to the metabolism of exogenous toxins in plants. The expression levels of down-regulated DEGs related to these two enzymes in the ST vs. RT comparison group were verified. The results were consistent with the transcriptome data (Figure 7 and Table 2), confirming the accuracy of the transcriptome results and providing a basis for the subsequent verification of key metabolic function genes.

### 2.8. The MDA Content and the Activity of Antioxidant Enzymes

**Figure 8:**
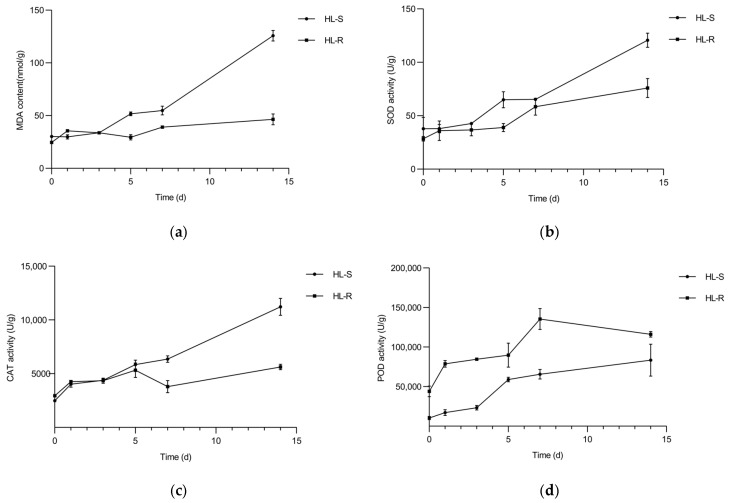
MDA content and activity of protective enzymes after treatment with cyhalofop-butyl in HL-S and HL-R populations. (**a**) The MDA content; (**b**) the activity of SOD; (**c**) the activity of CAT; (**d**) the activity of POD. Values are the mean ± SE of three biological replicates.

Following cyhalofop-butyl treatment, MDA content in the HL-S population increased continuously until plant death, whereas it remained relatively stable in the HL-R population throughout the experimental period (Figure 8a). Regarding SOD activity, no significant difference was observed between populations on days 1 and 3. On day 5, SOD activity in HL-S was significantly higher than that in HL-R. On day 7, no significant difference was found, but on day 14, SOD activity in HL-S was extremely significantly higher than in HL-R (Figure 8b). CAT activity did not differ significantly between the two populations on days 1, 3, and 5; however, on days 7 and 14, CAT activity in HL-S was extremely significantly higher than that in HL-R (Figure 8c). Notably, POD activity in HL-S was extremely significantly lower than that in HL-R at each time point (Figure 8d).

## 3. Discussion

The management of resistant barnyard grass has become a key issue that needs to be urgently addressed. Research has shown that barnyard grass has developed resistance to various herbicides, including penoxsulam, metamifop, and quinclorac [30]. Cyhalofop-butyl has been used for over 20 years and unavoidably falls into the high-risk category in resistance risk classification [31]. Significant differences in resistance mechanisms among barnyard grass populations to cyhalofop-butyl have been reported. Therefore, a comprehensive analysis of these resistance mechanisms is an important prerequisite for efficiently solving the problem of resistant barnyard grass.

### 3.1. The Resistance of Barnyard Grass to Cyhalofop-Butyl Is Related to Overexpression of ACCase Gene

The results showed that the *GR_50_* values of HL-S and HL-R to cyhalofop-butyl were 28.33 and 539.8 g a.i.ha^−1^, respectively. The RI of HL-R was 19.05-fold that of HL-S. Cross-resistance and multi-resistance assays indicated that the resistance indices of HL-R to propanil, metamifop, quinclorac, pyribenzoxim and penoxsulam were 5.14, 14.53, 6.21, 1.98 and 6.24-fold that of HL-S, respectively. These findings confirm that herbicide resistance in barnyard grass has become increasingly severe, consistent with previous surveys showing that the field application rates of cyhalofop-butyl have exceeded the recommended dosage more than 10-fold.

TSR is one of the resistance mechanisms to herbicide in weeds. For instance, the Asp2027Cys mutation confers resistance to cyhalofop-butyl, fenoxaprop-P-ethyl and pinoxaden in *E. crus-galli* [32]. Up-regulated expression of target enzyme genes is also a key factor in resistance in *Eleusine indica* and *E. crus-galli* to glyphosate and cyhalofop-butyl [9,33]. Additionally, multiple resistance in *E. crus-galli* to cyhalofop-butyl, penoxsulam and other herbicides is synergistically mediated by the Ile2041Thr mutation and overexpression of the *ACCase* [34]. In this study, TSR analysis indicated that no mutations were detected at any previously reported *ACCase* mutation loci in the resistant population HL-R compared to the susceptible population HL-S. However, an extremely significant difference was observed in the expression levels of the *ACCase* gene between the two populations. These findings demonstrate that the resistance of HL-R to cyhalofop-butyl is not associated with mutations in the coding regions of the target enzyme gene, but is closely linked to the overexpression of the *ACCase* gene. It should be noted that *E. crus-galli* is hexaploid; the qRT-PCR results reflect the combined expression of all *ACCase* homeologs, and future studies using homeolog-specific primers may reveal differential contributions of individual copies.

Moreover, NTSR mostly results in moderate or low-level resistance, with a few cases leading to high-level resistance, and even inducing cross-resistance to herbicides with different modes of action [18]. Driven by complex evolutionary pressures, NTSR exerts a more profound impact on weed management compared with TSR. NTSR is involved in herbicide absorption, translocation and metabolism in weeds. Herbicide metabolism in weeds is a complex process mediated by multiple gene families, including CYP450s, GSTs, GTs and ABC transporters [18,35]. This study showed that malathion and NBD-Cl significantly reduced the *GR_50_* of HL-R but had no effect on HL-S, providing evidence for the existence of NTSR in HL-R.

### 3.2. P450 and GST Genes as Key Regulators Mediate Resistant Processes

Transcriptomic analysis has become a pivotal approach for investigating NTSR mechanisms in weeds [36]. Screening herbicide resistance-related DEGs via transcriptomics and obtaining full-length sequences of target genes by aligning with genomic data have been widely adopted. For example, Yang et al. demonstrated that enhanced P450 activity contributes to cyhalofop-butyl resistance in a Chinese *E. crus-galli* population [37], and Jin et al. identified multiple metabolic genes, including GST and ABC transporters, associated with cyhalofop resistance [38]. In this study, metabolism-related down-regulated DEGs were selected. Based on differential expression significance and functional implications, one P450 gene and one GST gene (*CH01.636* and *BH02.4557*) were ultimately chosen for validation. The qRT-PCR validation results were consistent with the transcriptomic analysis, although the specific functions of these two genes require further research. Our findings of P450- and GST-related DEGs are consistent with previous research, further supporting the role of metabolic detoxification in NTSR.

### 3.3. POD Activity Was Related to Detoxification of Cyhalofop-Butyl in Resistant Barnyard Grass

Low levels of ROS act as signaling molecules in response to biotic or abiotic stresses and can interact with epigenetic modifiers and hormones to regulate plant development [39]. In contrast, excessive ROS can induce membrane lipid peroxidation, abnormal growth, or even cell death [40]. Plants possess an antioxidant defense system that can eliminate ROS to a certain extent: SOD converts O_2_- into H_2_O_2_, while CAT and POD reduce H_2_O_2_ to H_2_O [41]. In this study, cyhalofop-butyl treatment resulted in a continuous increase in MDA content in HL-S until plant death, while that in HL-R remained relatively stable throughout the experimental period. This observation indicated that after exposure to cyhalofop-butyl, HL-S experienced sustained ROS accumulation, causing severe cellular damage and ultimately plant death. Conversely, ROS in HL-R were likely promptly cleared by POD. Notably, SOD and CAT activities in HL-R were consistently lower than those in HL-S. This finding suggests that POD activity is involved in the mechanism responsible for the resistance of HL-R to cyhalofop-butyl.

### 3.4. Limitations and Perspectives

Several limitations of this study should be acknowledged. First, our findings are based on a single resistant population (HL-R) from Hulin, Heilongjiang; thus, the generality of the observed resistance mechanisms to other barnyard grass populations requires further investigation. Second, although transcriptome analysis identified two candidate genes (*CH01.636* and *BH02.4557*) associated with P450 and GST pathways, their specific roles in cyhalofop-butyl detoxification have not been functionally validated. Future studies should employ heterologous expression (e.g., in yeast or Arabidopsis) or gene silencing (e.g., RNAi or CRISPR/Cas9) to confirm the function of these genes. Third, the involvement of POD activity in resistance was inferred from enzyme activity assays; direct evidence of ROS scavenging by POD in resistant plants is needed. Metabolomics approaches could also be applied to identify the specific degradation products of cyhalofop-butyl in HL-R, providing direct evidence of enhanced metabolic detoxification. Overall, our study provides a foundation for further mechanistic dissection of multi-mechanism synergistic resistance in barnyard grass.

## 4. Materials and Methods

### 4.1. Materials and Treatment

The barnyard grass seeds were collected from Hulin (45°58′ N, 132°54′ E), Heilongjiang Province, China. The HL-R population was gathered from fields where cyhalofop-butyl had been continuously applied for many years, while the HL-S population was collected from wastelands that had never been treated with herbicides. Seeds were stored at 4 °C and then soaked in water at 25 °C for 24 h before sowing. Plants were grown in a greenhouse under controlled conditions: 28/22 °C day/night, and 60–70% relative humidity. The herbicides used in this experiment are shown in Table 3.

The plastic pots (9 cm × 9 cm × 10 cm) were filled with equal weights of seedling substrate (peat, vermiculite and perlite in a 3:1:1 ratio). A uniform number of barnyard grass seeds was sown in each pot, covered with 0.5–1.0 cm of seedling substrate, gently pressed, thoroughly watered, placed in a greenhouse for cultivation, and watered as needed to maintain soil moisture at approximately 60–70% field capacity. When plantlets reached the one-leaf stage, they were thinned to 20 plants per pot.

### 4.2. The Whole-Plant Dose–Response Bioassay

When plantlets reached the three- to four-leaf stage, herbicides were applied at varying dose (Table 3) using STC01Z walking spray tower (Research Equipment Co., Beijing, China) with a spray volume of 450 L/ha and pressure of 0.275 MPa. Clear water was sprayed as the untreated control. Each treatment consisted of three biological replicates. Twenty-one days after application, the above-ground parts were collected, and the fresh weight was measured. According to the fresh weight inhibition rate, the *GR_50_* was calculated using the four-parameter log-logistic model (GraphPad Prism 8.0.1) according to the following formula [42]:

*Y* = *C* + (*D* − *C*)/[1 + (*X*/*GR*_50_)]^*b*, (1)

where *Y* is the fresh weight under treatment, D is the upper limit of dosage, C is the lower limit of dosage, b is the slope at the *GR_50_*.

### 4.3. ACCase Gene Sequencing

*E. crus-galli* plants were cultured to three- to four-leaf stage. Ten individual plants from each population were sampled for DNA extraction. These plants were not pre-exposed to cyhalofop-butyl or any other herbicide, ensuring that the detected mutations represent the frequency in the HL-R population. Tender leaves were collected, and DNA was extracted according to the instructions of the Sangon Biotech (Shanghai, China) Plant DNA Extraction Kit, and the quality was assessed. To detect the 7 reported amino acid mutations in the carboxyltransferase (CT) domain of *ACCase*, the primers *ACCase-F* (5′-CTCTTATATACTTGGCAGCAAACTC-3′) and *ACCase-R* (5′-TTCCCAATCTACAACTTTCTTAATC-3′) were used. The PCR mixture (25 μL) contained 1 μL of template DNA, 1 μL of 10 μM forward primer, 1 μL of 10 μM reverse primer, 1 μL of 10 μM dNTP mix, 2.5 μL of 10× Taq Buffer (with MgCl_2_), 0.2 μL of 5 U/μL Taq enzyme, and ddH_2_O. The reaction conditions were as follows: predenaturation at 95 °C (5 min), denaturation at 94 °C (30 s), annealing at 63 °C (with a reduction of 0.5 °C per cycle) for 30 s, and extension to 72 °C (30 s)—10 cycles. Then denaturation at 95 °C (30 s), annealing at 58 °C (30 s), and extension to 72 °C (30 s)—30 cycles. Extend at 72 °C for another 10 min. PCR products were detected by 1% agarose gel electrophoresis, purified and recovered using a Sangon Biotech (Shanghai, China) SanPrep columnar DNA gel recovery kit. Sequencing was performed, and results were analyzed using Sequencing Analysis v5.2.0. Base and amino acid sequences of the functional regions of *ACCase* CT in the two populations were compared and analyzed using SeqMan 7.1.0. Given the hexaploid nature of *E. crus-galli*, primers were designed to amplify all homeologous copies; chromatograms were inspected for overlapping peaks indicative of sequence heterogeneity, and none were observed.

### 4.4. ACCase Gene Expression

For *ACCase* gene expression analysis, plants were sprayed with cyhalofop-butyl (120 g a.i.ha^−1^). Tender leaves (3 g) were collected at 0, 1, 2, 3 and 5 days after application. RNA was extracted according to the instructions of the Total RNA Extractor (Trizol, Thermo Fisher Scientific, Waltham, MA, USA), and RNA integrity was verified by agarose gel electrophoresis. Using the full length *ACCase* sequence of *Echinochloa phyllopogon* (Stapf) Koss as a template, the *EcActin* primer sequences, *EcActin-F* (GTGCTGTTCCAGCCATCGTTCAT) and *EcActin-R* (CTCCTTGCTCATACGGTCAGCAATA), target gene primer sequence *ACCase-F* (5′-AGAATGCTGCTTAGACGCAATAC-3′) and *ACCase-R* (5′-GGATAACCAACCACCTGACAAC-3′) were designed using Primer Premier 5.0 and synthesized by Sangon Biotech (Shanghai, China) Co., Ltd. The reaction mixture (20 μL) contained 10 μL of 2× SybrGreen qPCR Master Mix, 0.4 μL of 10 μM forward primer, 0.4 μL of 10 μM reverse primer, 2 μL of cDNA, and 7.2 μL of ddH_2_O. The PCR cycling conditions were as follows: predenaturation at 95 °C (3 min), denaturation at 95 °C (15 s), and annealing at 60 °C (30 s)—45 cycles. The experiment was performed on an ABI 7500 real-time PCR system (Applied Biosystems, Waltham, MA, USA). The relative expression of *ACCase* gene was calculated using the 2^−(ΔΔCt)^ formula [43], and multiple comparisons were performed using Duncan’s test in DPS 7.05 statistical analysis software.

### 4.5. Malathion and NBD-Cl Verification

To verify whether resistance to cyhalofop-butyl is related to P450 and GST, experiments were conducted using the P450 inhibitor malathion and the GST inhibitor NBD-Cl. At the three- to four-leaf stage of *E. crus-galli*, malathion (1000 g a.i.ha^−1^) and NBD-Cl (270 g a.i.ha^−1^) [44] were sprayed 2 and 48 h before cyhalofop-butyl (15–1920 g a.i.ha^−1^) application, respectively, using the laboratory sprayer as described above.

### 4.6. RNA-Seq Analysis

#### 4.6.1. RNA Extraction and cDNA Library Construction

At the three- to four-leaf stage of *E. crus-galli*, plants were sprayed with the recommended dose (120 g a.i.ha^−1^) of cyhalofop-butyl. Tender leaves (0.1 g) were collected at 0 and 48 h after application, frozen in liquid nitrogen, and stored at −80 °C. Total RNA was extracted using the RNA kit (Sangon Biotech (Shanghai, China), B511311) following the manufacturer’s instructions. Extracted RNA was subjected to quality control, including concentration and fragment length detection using 1% agarose gel electrophoresis, NanoDrop 2000 ultramicro spectrophotometer (Thermo Fisher Scientific, Waltham, MA, USA) and Agilent 2100 Bioanalyzer (Agilent Technologies, Santa Clara, CA, USA). A cDNA library was constructed using the NEBNext Ultra RNA library Prep Kit (New England Biolabs, Ipswich, MA, USA) for Illumina and sequenced on an Illumina Hiseq 4000 platform (Agilent Technologies, Santa Clara, CA, USA). Three biological replicates per treatment were sequenced.

#### 4.6.2. Data Preprocessing

Raw sequencing data were quality-assessed using FastQC (v0.12.1) and then trimmed for quality using fastp to obtain clean reads. Clean reads were aligned to *E. crus-galli* genome sequence [29] using HISAT2 (v2.2.1). Alignment results were statistically analyzed using RSeQC (v5.0.1).

#### 4.6.3. Differential Expression Analysis and DEG Functional Enrichment

DEGs were identified using DESeq2 (v1.38.3) with screening criteria of *q* < 0.05 and |log_2_(fold change)|> 1.0 [45]. GO enrichment analysis was performed using GOSeq (v1.50.0) and TopGov 1.22. The KEGG database was used for KEGG pathway analysis of DEGs, and a hypergeometric distribution test was used to identify significantly enriched (*p* < 0.01) GO terms and KEGG pathways. 

### 4.7. qRT-PCR to Verify DEGs

This study focused on genes related to metabolism. From the P450 and the GST family genes, *CH01.636* and *BH02.4557* were selected for expression level verification. The method is described in Section 4.4, and the primer sequences were: *CH01.636*-F (5′-CCAACCTGTGCGACTACCTG-3′) and *CH01.636*-R (5′-AGAGCAGCACGGCAATCAT-3′), *BH02.4557*-F (5′-TGTGAGTCCCTTGTCATTGTTG-3′) and *BH02.4557*-R (5′-ACCTCGCCCTCTGTCCAGT-3′). qRT-PCR was performed on three biological replicates (each from different plants) and three technical replicates per sample. The 2^−(ΔΔCt)^ method was used for relative quantification, with EcActin as the internal control.

### 4.8. Determination of MDA and Activity of SOD, CAT and POD

Samples were collected at 0, 1, 3, 5, 7 and 14 days after spraying cyhalofop-butyl (120 g a.i.ha^−1^) at three- to four-leaf stage and stored at −80 °C. The MDA (532 nm) content was determined by the TBA colorimetric method [46], SOD (450 nm, U/g FW) activity was measured by WTS-8 method [47], CAT (240 nm) activity by the UV colorimetric method [47], and POD (470 nm) activity by guaiacol colorimetric method [47], using the SpectraMax ABS plus reader (Molecular Devices, San Jose, CA, USA). Three biological replicates were used per time point, and each sample was measured in triplicate.

## 5. Conclusions

The high resistance of HL-R (collected from Hulin, Heilongjiang) to cyhalofop-butyl is attributed to the synergistic effect of *ACCase* overexpression (TSR) and P450/GST-mediated metabolic detoxification (NTSR). Additionally, this population exhibits broad-spectrum herbicide resistance, but pyribenzoxim presents a relatively low resistance risk, and may serve as a promising alternative rotational herbicide for the management of herbicide-resistant barnyard grass. These findings provide a theoretical basis for elucidating the molecular mechanism underlying multi-mechanism synergistic resistance in barnyard grass, and also offer practical guidance for the scientific control of resistant barnyard grass and rational rotation of herbicides in cold-region rice fields of Northeast China. Future studies are required to functionally verify the candidate genes to clarify their specific mechanisms of action.

## Figures and Tables

**Table 1:** The influence of malathion and NBD-Cl on cyhalofop-butyl resistance in HL-S and HL-R.

Populations	Treatments	*GR_50_* (g a.i.ha^−1^)	RI
HL-S	cyhalofop-butyl	28.3	1
	cyhalofop-butyl + malathion	30.8	1.09
	cyhalofop-butyl + NBD-Cl	28.0	0.99
HL-R	cyhalofop-butyl	539.8	19.05
	cyhalofop-butyl + malathion	109.2	3.85
	cyhalofop-butyl + NBD-Cl	139.0	4.91

**Table 2:** Transcriptome sequencing results of DEGs.

Gene ID	CK vs. RT			CK vs. ST			ST vs. RT		
	*p*	q	Result	*p*	q	Result	*p*	q	Result
*CH01.636*	2.03 *×* 10^−174^	2.13 *×* 10^−171^	down	3.78 *×* 10^−72^	7.03 *×* 10^−70^	down	1.94 *×* 10^−15^	4.10 *×* 10^−13^	down
*BH02.4557*	2.20 *×* 10^−83^	3.94 *×* 10^−81^	down	1.62 *×* 10^−36^	8.23 *×* 10^−35^	down	5.30 *×* 10^−11^	5.61 *×* 10^−09^	down

**Table 3:** Information on the herbicides used in the resistance determination of *Echinochloa crus-galli*.

Herbicide	Registration Code	Dose (g a.i.ha^−1^)
10% cyhalofop-butyl OD	PD20160440	15.0, 30.0, 60.0, 120.0 *, 240.0, 480.0, 960.0, 1920.0
34% propanil EC	PD20210496	175.3, 350.6, 701.3, 1405.5 *, 2805.0, 5610.0, 11,220.0
10% metamifop EC	PD20183270	17.3, 34.7, 69.4, 138.8 *, 277.5, 555.0, 1110.0
50% quinclorac WP	PD20080973	18.8, 37.5, 75.0, 150.0, 300.0 *, 600.0, 1200.0, 2400.0
10% pyribenzoxim OD	PD20182251	1.9, 3.8, 7.5, 15.0 *, 30.0
25 g/L penoxsulam OD	PD20170671	2.8, 5.6, 11.3, 22.5 *, 45.0, 90.0, 180.0 360.0

* is the recommended field dose.

## Data Availability

The original contributions presented in this study are included in the article/Supplementary Materials. Further inquiries can be directed to the corresponding author.

## Supplementary Materials

The following supporting information can be downloaded at: https://www.mdpi.com/article/10.3390/plants15121908/s1. Table S1: Summary of RNA-seq read alignment statistics. Table S2: Complete list of differentially expressed genes (CK vs. ST). Table S3: Complete list of differentially expressed genes (ST vs. RT). Table S4: Complete list of differentially expressed genes (CK vs. RT). Table S5: GO enrichment results for DEGs (CK vs. ST). Table S6: GO enrichment results for DEGs (ST vs. RT). Table S7: GO enrichment results for DEGs (CK vs. RT). Table S8: KEGG pathway enrichment results (CK vs. ST). Table S9: KEGG pathway enrichment results (ST vs. RT). Table S10: KEGG pathway enrichment results (CK vs. RT).
